# Management of tracheostomy-related emergencies: An audit of junior doctors' knowledge and skills

**DOI:** 10.1186/1749-8090-10-S1-A278

**Published:** 2015-12-16

**Authors:** Georgina Findlay, Yasmin Abbas

**Affiliations:** 1 Otolaryngology department, West Middlesex University Hospital, London, TW7 6AF, UK

## Background/Introduction

The 2014 National Confidential Enquiry into Patient Outcome and Death(NCEPOD) highlighted hospital tracheostomy care as a significant patient safety issue (1) Tracheostomy-related complications occur frequently in ward patients, and are potentially life-threatening. (2) Rapid recognition and management of blocked or displaced tracheostomy tubes is essential. According to Intensive Care Society guidelines "All staff working in clinical locations where tracheostomy patients are managed must be competent to assess and initiate management in the event of airway emergency occurring". (3) Junior doctors are often the first called to manage such patient deteriorations.

## Aims/Objectives

The aim of this study was to ascertain junior doctors' confidence and competence levels in the basic management of tracheostomy patients who develop airway problems. The overall objective was to ensure that junior doctors can recognise and rapidly instigate effective resuscitative measures.

## Method

Questionnaires were circulated to junior doctors working on general medical and surgical wards, and the Accident and Emergency department. Doctors rated their confidence in recognising airway obstruction in tracheostomy patients and in initiating management of this. 8 multiple choice questions (MCQs) based on the Resuscitation Council's tracheostomy algorithm were also completed. Following this, teaching sessions and practical workshops were organised and doctors repeated the questionnaire.

## Results

38 questionnaires were completed and returned by junior doctors. 24 (63.2%) had previously cared for patients with a tracheostomy. 8 (21.1%) felt confident in recognising airway obstruction and 6 (15.8%) felt confident or very confident in initiating initial management for a blocked or displaced tracheostomy. The mean score of 8 MCQ questions was 45.1%.

After the teaching sessions, repeat questionnaires confirmed significantly improved confidence levels, and dramatically improved MCQ scores.

## Discussion/Conclusion

The management of airway obstruction in patients with tracheostomies is poorly understood by junior doctors. This poses a significant patient safety concern. Teaching workshops with practical demonstrations are effective measures to improve doctors' confidence and management of emergencies.

